# Supplementary material for: AffyMAPSDetector: a software tool to characterize Affymetrix GeneChip™ expression arrays with respect to SNPs
Source: BMC Bioinformatics. 2007 Jul 30;8:276. doi: 10.1186/1471-2105-8-276 (PMC1959249; doi:10.1186/1471-2105-8-276)
Supplement: Additional file 13 — AffyMAPSDetector v1 source code. AffyMAPSDetector source code. [file 1471-2105-8-276-S13.zip › AffyMAPSDetector_v1_src/doc/affychip/snpdetector/model/SnpMatcher.FileClosingShutdownHook.html]

SnpMatcher.FileClosingShutdownHook


|  |  |  |  |
| --- | --- | --- | --- |
| **Overview** \| **Package** \| **Class** \| **Use** \| **Tree** \| **Deprecated** \| **Index** \| **Help** | | |  |
| **PREV CLASS**   NEXT CLASS | **FRAMES**    **NO FRAMES**     **All Classes** |  |  |
| SUMMARY: NESTED \| FIELD \| CONSTR \| METHOD | DETAIL: FIELD \| CONSTR \| METHOD |  |  |


---


## affychip.snpdetector.model Class SnpMatcher.FileClosingShutdownHook

```
java.lang.Object
  java.lang.Thread
      affychip.snpdetector.model.SnpMatcher.FileClosingShutdownHook
```

**All Implemented Interfaces:**: java.lang.Runnable

**Enclosing class:**: SnpMatcher

---

public class **SnpMatcher.FileClosingShutdownHook** extends java.lang.Thread

Shudown hook that would ensure that if the program terminates abnormally
then all opened data files, to which data is written, are properly closed
without loosing any data that has not yet been commited to disk.

Title: File Manager

Description: Split probe data file generated by dChip

Copyright: Copyright (c) 2004

Company: George Mason University

**Version:**
:   1.1

**Author:**
:   Sunita Kumari

---

**Field Summary**

**Fields inherited from class java.lang.Thread**

`MAX_PRIORITY, MIN_PRIORITY, NORM_PRIORITY`


**Constructor Summary**

`SnpMatcher.FileClosingShutdownHook()`

`SnpMatcher.FileClosingShutdownHook(java.io.Writer[] outputFile,
java.io.Reader[] inputFile)`


|  |  |
| --- | --- |
| **Method Summary** | |
| `void` | `run()` |

**Methods inherited from class java.lang.Thread**

`activeCount, checkAccess, countStackFrames, currentThread, destroy, dumpStack, enumerate, getContextClassLoader, getName, getPriority, getThreadGroup, holdsLock, interrupt, interrupted, isAlive, isDaemon, isInterrupted, join, join, join, resume, setContextClassLoader, setDaemon, setName, setPriority, sleep, sleep, start, stop, stop, suspend, toString, yield`

**Methods inherited from class java.lang.Object**

`clone, equals, finalize, getClass, hashCode, notify, notifyAll, wait, wait, wait`

**Constructor Detail**

### SnpMatcher.FileClosingShutdownHook

```
public SnpMatcher.FileClosingShutdownHook(java.io.Writer[] outputFile,
                                          java.io.Reader[] inputFile)
```

---


### SnpMatcher.FileClosingShutdownHook

```
public SnpMatcher.FileClosingShutdownHook()
```


**Method Detail**

### run

```
public void run()
```


---


|  |  |  |  |
| --- | --- | --- | --- |
| **Overview** \| **Package** \| **Class** \| **Use** \| **Tree** \| **Deprecated** \| **Index** \| **Help** | | |  |
| **PREV CLASS**   NEXT CLASS | **FRAMES**    **NO FRAMES**     **All Classes** |  |  |
| SUMMARY: NESTED \| FIELD \| CONSTR \| METHOD | DETAIL: FIELD \| CONSTR \| METHOD |  |  |


---
